# CD80 on Human T Cells Is Associated With FoxP3 Expression and Supports Treg Homeostasis

**DOI:** 10.3389/fimmu.2020.577655

**Published:** 2021-01-08

**Authors:** Blagoje Soskic, Louisa E. Jeffery, Alan Kennedy, David H. Gardner, Tie Zheng Hou, Neil Halliday, Cayman Williams, Daniel Janman, Behzad Rowshanravan, Gideon M. Hirschfield, David M. Sansom

**Affiliations:** ^1^ Institute of Immunity and Transplantation, Division of Infection & Immunity, University College London, Royal Free Hospital, London, United Kingdom; ^2^ Institute of Metabolism and Systems Research, College of Medical and Dental Sciences, University of Birmingham, Edgbaston, Birmingham, United Kingdom; ^3^ School of Immunity and Infection, College of Medical and Dental Sciences, University of Birmingham, Edgbaston, Birmingham, United Kingdom; ^4^ Toronto Centre for Liver Disease, Toronto General Hospital, Toronto, ON, Canada

**Keywords:** CD80 and CD86, CD28 costimulation, CTLA-4, T cell activation, Treg

## Abstract

CD80 and CD86 are expressed on antigen presenting cells (APCs) and their role in providing costimulation to T cells is well established. However, it has been shown that these molecules can also be expressed by T cells, but the significance of this observation remains unknown. We have investigated stimuli that control CD80 and CD86 expression on T cells and show that in APC-free conditions around 40% of activated, proliferating CD4^+^ T cells express either CD80, CD86 or both. Expression of CD80 and CD86 was strongly dependent upon provision of CD28 costimulation as ligands were not expressed following TCR stimulation alone. Furthermore, we observed that CD80^+^ T cells possessed the hallmarks of induced regulatory T cells (iTreg), expressing Foxp3 and high levels of CTLA-4 whilst proliferating less extensively. In contrast, CD86 was preferentially expressed on INF-γ producing cells, which proliferated more extensively and had characteristics of effector T cells. Finally, we demonstrated that CD80 expressed on T cells inhibits CTLA-4 function and facilitates the growth of iTreg. Together these data establish endogenous expression of CD80 and CD86 by activated T cells is not due to ligand capture by transendocytosis and highlight clear differences in their expression patterns and associated functions.

## Introduction

The activation of CD4^+^ T cells is dependent upon two signals provided by antigen presenting cells (APCs). The first signal, which determines the specificity of response, is generated by TCR recognition of antigen displayed by MHC II. In addition, in response to inflammatory signals, APCs upregulate the costimulatory ligands CD80 and CD86, which then interact with the CD28 receptor expressed on T cells. This costimulatory “second signal” lowers the threshold of T cell activation (1), promotes T cell survival (2) and enhances production of cytokines (3), particularly the T cell growth factor IL-2 (4, 5).

Accordingly, control of the CD28 signal represents a key immune checkpoint, ensuring that T cell responses are initiated to antigens recognised on APC expressing costimulatory ligands. Availability of CD28 ligands is regulated by the inhibitory receptor CTLA-4 (6–8). Since CTLA-4 binds to both CD80 and CD86 with a higher affinity than CD28 (9), CTLA-4 is able to compete with CD28 for CD80/86 binding. In addition, CTLA-4 physically removes CD80 and CD86 from APCs by the process of trans-endocytosis, impairing the ability of APCs to activate T cells (10, 11). Therefore, CTLA-4 serves as a checkpoint in the prevention of autoimmunity. Ligand removal by CTLA-4 fits well with the requirement for CTLA-4 on regulatory T cells (Treg), however other functions of CTLA-4 have been extensively debated (12). Whilst the costimulatory functions of CD80 and CD86 in the context of antigen presenting cells are widely appreciated, both ligands can also be expressed on activated T cells (13–16). However, CD80 and CD86 distribution on different T cell subsets and the significance of ligand expression in this setting remain unclear. Moreover, since both CD28 and CTLA-4 can promote transfer of ligands to T cells it is not yet established whether CD80 and CD86 are intrinsically expressed by T cells or are acquired from APCs (10, 17–19).

To address these issues, we examined whether CD80 and CD86 were endogenous to activated T cells, as well as exploring the stimuli that influenced their expression. Here we show that in APC-free conditions where ligand acquisition is not possible, around 40% of activated, proliferating CD4^+^ T cells express either CD80, CD86 or both. Expression of CD80 and CD86 was dependent upon provision of CD28 costimulation as ligands were not expressed following TCR stimulation alone. We observed that CD86 was preferentially expressed on IFN-γ producing cells, which had proliferated extensively and had characteristics of effector T cells. In contrast, we observed that CD80^+^ T cells possessed the hallmarks of induced regulatory T cells, expressing Foxp3 and high levels of CTLA-4 whilst proliferating less extensively. We also found that CD80 expressed on Tregs acts as an intrinsic ligand for CTLA-4, thus modulating CTLA-4 and enhancing Treg proliferation *via* CD28. Together these data establish endogenous expression of CD80 and CD86 by human T cells and highlight clear differences in their expression patterns and likely functions.

## Materials and Methods

### Cell Culture

Chinese hamster ovary cells (CHO) transduced with human CD80, CD86 and FcRγII (CD32) were cultured in DMEM (Invitrogen, Paisley, UK) supplemented with 10% FBS (Biosera, Uckfield, UK), 2mM L-glutamine (Sigma, Gillingham, UK) and 1% penicillin and streptomycin (Invitrogen). Cells were cultured at 37°C in 5% CO_2_ and passaged every two to three days by trypsinisation. T cells were cultured in RPMI (Invitrogen) supplemented with 10% FBS (Biosera, Uckfield, U.K.), 2mM L-glutamine (Sigma) and 1% penicillin and streptomycin (Invitrogen) at 37°C/5% CO_2_.

### Cell Isolation

Peripheral blood mononuclear cells (PBMCs) were isolated using Ficoll-Paque PLUS (GE healthcare, Buckingham, UK) density gradient centrifugation. CD4^+^ CD25^-^ T cells were purified using EasySep^®^ CD4^+^ CD25^-^ enrichment kit (StemCell Technologies, Meylan, France) according to manufacturer’s instructions. Monocytes were purified using EasySep^®^ monocyte enrichment kit (StemCell Technologies, Meylan, France) according to the manufacturer’s instructions.

### T Cell Activation Assays

CD4^+^ CD25^-^ T cells were labelled using Cell Trace™ Violet Proliferation kit (Invitrogen). Following 20 min of the incubation with 2.5µM Cell Trace™ Violet dye at 37°C, dye was quenched with excess RPMI and washed ready for use. A total of 1 × 10^5^ T cells were stimulated in round bottom 96 well plates for 5 days (unless otherwise stated).

Beads stimulation: T cells were stimulated with anti-CD3/anti-CD28 human T-expander Dynabeads^®^ (Invitrogen) at 1 bead to 4 T cell ratio.

CHO cell stimulation: CHO-CD80/86 or CHO-FcR cells were fixed in 1 ml of 0.025% glutaraldehyde. CD4^+^ CD25^-^ T cells were stimulated with 0.5 µg/ml of anti-CD3 (clone OKT3) in the presence of fixed CHO-CD80/86 at 1:5 CHO:T cell ratio. Where indicated anti-CD28 (clone 9.3) was added at 0.5 µg/ml.

Where indicated, activations were supplemented with 100 U/ml IL-2 (PeproTech), 1 ng/ml TGF-β (R&D Systems, Abingdon, UK), 10 ng/ml IL-12 (PeproTech) and 25 nM Torin 1 (Tocris).

### Treg Expansion

CD4^+^ CD25^+^ T cells were positively selected from the CD4^+^ fraction with anti-CD25 antibody (StemCell) according to manufacturer’s instructions and cultured in a 24 well plate. T cells were stimulated with anti-CD3/anti-CD28 beads at 1:1 ratio in the presence of 100nM Rapamycin (LKT Laboratories, Minnesota). Two days later, cultures were supplemented with 1,000 IU/ml IL-2, which was repeated every 2–3 days. Stimulation beads were magnetically removed 7 days following stimulation and restimulated with anti-CD3/anti-CD28 beads at 1:1 ratio in the presence of IL-2 and rapamycin. Seven days after the second stimulation beads were magnetically removed and T cells were rested for two days, after which cells were restimulated with beads or CHO-CD86.

### Intracellular Cytokine Staining

Prior to the cytokine staining, cells were sorted based upon CD80 and CD86 expression using a Moflow Cell Sorter and restimulated with 50 ng/ml phorbol myristate acetate (PMA) (Sigma) and 1 µM Ionomycin (Sigma) for 5 h in the presence of 10 µg/ml of Brefeldin A (Sigma) at 37°C. After 5 h cells were fixed with 3% Paraformaldehyde (PFA) for 15 min at room temperature, and permeabilised by washing with 0.1% saponin in PBS.

### Flow Cytometry

The following antibodies were used: CD80 (L307.4; BD Biosciences), CD86 (FUN-1; BD Biosciences), CD86 (IT2.2, BioLegend), CD25 (BC96; eBioscience), FoxP3 (236A/E7; eBioscience), CTLA-4 (BNI3; BD Biosciences), IFN-γ (B27; BD Biosciences), CD3 (UCHT1; BD Biosciences), CD4 (SK3; BD Biosciences), CD45RA (HI100; eBioscience), CD38 (HIT2; BD Biosciences) and HLA-DR (G46-6; BD Biosciences). For surface marker staining, cells were washed and re-suspended in 50 µl of FACS buffer (PBS and 2% Goat serum) containing antibodies conjugated with fluorochromes. Reactions were incubated for 30 min on ice. Cycling CTLA-4 was stained for 30 min at 37°C. For intracellular staining of FoxP3, a FoxP3 staining kit (eBioscences) was used according to the manufacturer’s instructions. Flow cytometry data were analysed by FlowJo (TreeStar, Ashland, Oregon, USA).

### CD86 Transendocytosis Assays

CD86GFP transfer from CHO cells expressing CD86-GFP into T cell was measured by flow cytometry as previously described [27] with the modification that CHO-CD86GFP cells were incubated together with T cells for 6 h in 96-well plates at 37°C at 1:1 ratio. Cultures were treated with 20nM Bafilomycin A1 (Sigma) to prevent ligand degradation.

### Statistical Analysis

Statistical analysis was performed using R version 3.6.1.

## Results

### CD80 and CD86 Are Expressed on Activated Treg and Tconv

**Figure 1:**
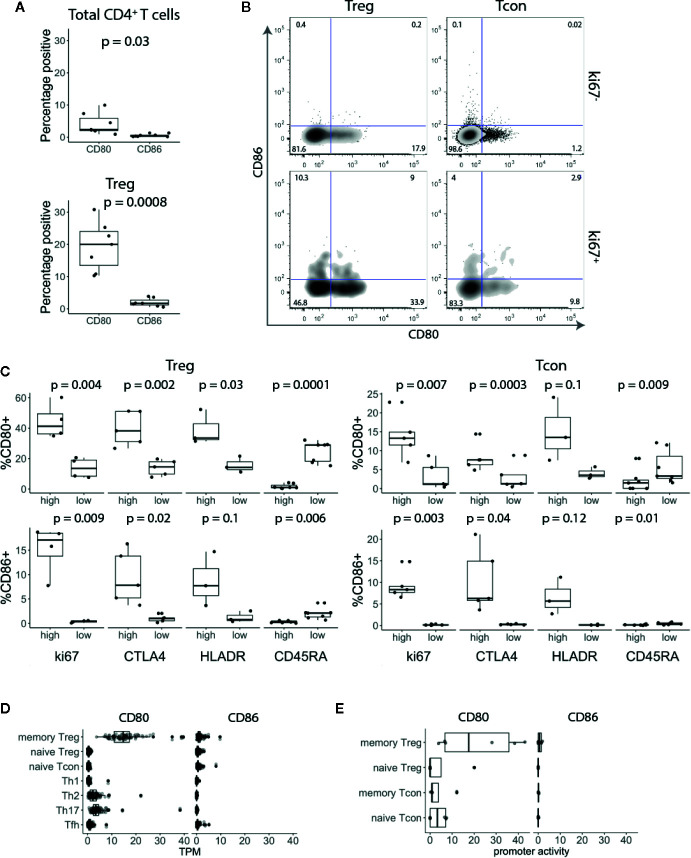
CD80 and CD86 are expressed on human *in vivo* activated conventional and regulatory T cells. **(A)** Graphs show percentage of CD80^+^ and CD86^+^ T cells across seven donors. **(B)** Cells were gated on CD3^+^ CD4^+^ T cells and based on FoxP3 expression divided into Treg and Tcon. Cells were further gated on Ki-67 and analysed for CD80 and CD86 expression. **(C)** Graphs show percentage of CD80^+^ and CD86^+^ Tcon and Treg expressing either high or low levels of Ki-67, CTLA-4, HLA-DR and CD45-RA. **(D**, **E)** Boxplots show CD80 and CD86 RNA expression levels **(D)** and promoter activity **(E)**. Data were downloaded from DICE and FANTOM5 databases respectively. TPM—transcripts per million. All p-values were calculated using unpaired two-tailed T-test.

To investigate CD80 and CD86 expression on T cells, we isolated peripheral blood mononuclear cells (PBMCs) from healthy blood donors and stained for CD80 and CD86 along with markers for naïve T cells (CD45RA) and regulatory T cells (Treg) (FoxP3) combined with T cell activation and proliferation markers (HLA-DR and Ki-67). *Ex vivo*, a small fraction of total CD3^+^ CD4^+^ T cells expressed CD80 or CD86 (**Figure 1A**). However, around 20% of Tregs expressed CD80, while less than 1% of them expressed CD86 (**Figure 1A**). Furthermore, we observed that CD80 was expressed on Tregs that predominantly expressed low levels of CD45RA and high levels of HLA-DR, Ki-67 and CTLA-4 (**Figures 1B, C**). CD86 was also expressed on activated Tregs (**Figure 1B**) however the percentage of CD86^+^ Tregs was significantly lower compared to CD80^+^ Tregs (**Figures 1A, B**). Similarly, we also observed that both CD80 and CD86 were expressed on activated conventional T cells (Tconv) (**Figure 1C**).

To validate these results, we assessed CD80 and CD86 RNA expression levels in publicly available data using the DICE study which profiled immune cell populations from blood on more than 90 donors (20). Consistent with our flow cytometry data, CD80 was almost exclusively expressed on Tregs with memory phenotype (**Figure 1D**). In addition, we examined the promoter activity in the FANTOM5 database (https://fantom.gsc.riken.jp/) and observed the same pattern, suggesting that CD80 is actively expressed by memory Tregs (**Figure 1E**). Overall, we concluded that CD80 and CD86 are expressed by a subset of blood CD4^+^ T cells with a distinct bias for CD80 expression on FoxP3^+^ T cells.

### CD28 Costimulation Up-Regulates CD80 and CD86 on Activated CD4^+^ T Cells

**Figure 2:**
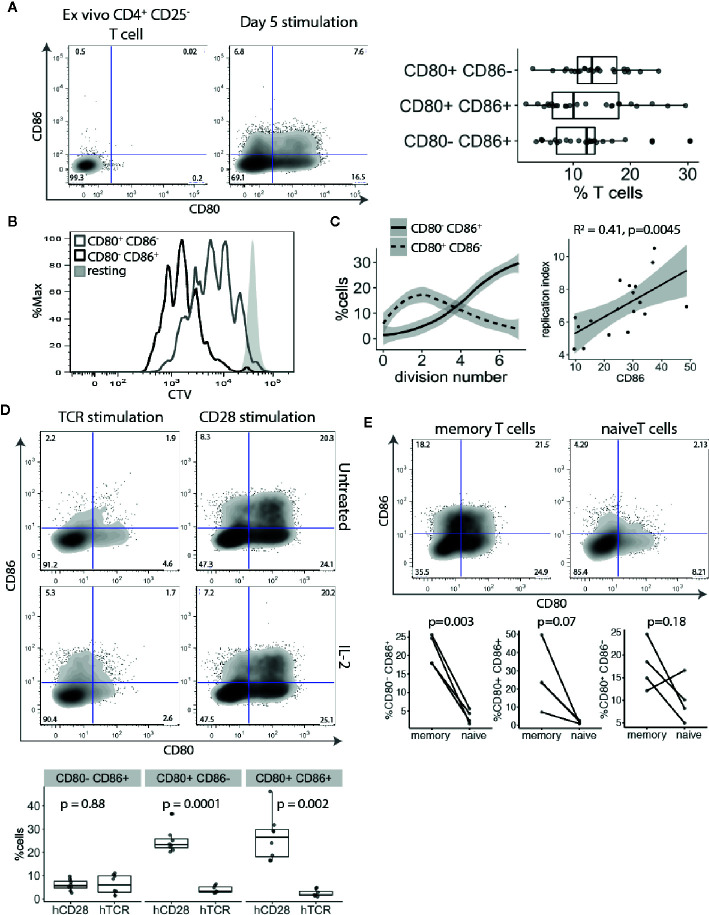
CD80 and CD86 are upregulated on human T cell activation. **(A)** Cell trace Violet labelled CD4^+^CD25^-^ T cells were stimulated for five days with anti-CD3/anti-CD28 coated beads and analysed for CD80 and CD86 expression on dividing cells. Representative FACS plots of CD80 and CD86 expression before stimulation and five days following stimulation. Graph shows the percentage of divided T cells expressing either CD80, CD86 or both across multiple donors. **(B)** Histogram shows proliferative capacity of CD80^+^ and CD86^+^ T cells. **(C)** Left graph shows the percentage of CD80^+^CD86^-^ and CD80^-^CD86^+^ T cells across different divisions. Right graph shows correlation between percentage of CD86^+^ T cells and replication index. P-value and R^2^ from linear regression model. **(D)** Cell trace Violet labelled CD4^+^CD25^-^ T cells were stimulated with 0.5 μg/ml anti-CD3 or 0.5 μg/ml anti-CD28 cross linked with CHO-FcR-expressing cells and analysed for CD80 and CD86 expression on dividing cells. Representative FACS plots and pooled data are shown for percentage of CD80 and CD86 expressing cells in divided cells. Cultures were supplemented with 100 U/ml of IL-2 as indicated. **(E)** Graphs show the percentage of stimulated and dividing naive and memory T cells expressing CD80, CD86 or both. P-values were calculated using paired two-tailed T-test.

The above transcriptional data suggest that CD80 is actively transcribed by Treg. However, to rule out any additional ligand transfer from interacting APCs, we used an APC-free stimulation system. CTV-labelled CD4^+^CD25^-^ T cells were stimulated with anti-CD3/anti-CD28 antibody-coated beads for five days and examined for CD80 and CD86 expression. This revealed that similar proportions of proliferating T cells stimulated in this APC-free condition expressed CD80, CD86 or both (**Figure 2A**). Since there is no other source of CD80 or CD86 in this system we concluded that expression was a T cell intrinsic response to stimulation. Moreover, given that all the cells analysed were gated on dividing cells, those cells not expressing CD80 or CD86 were not due to lack of activation. Next, we compared CD80 and CD86 expressing T cells for their proliferative capacity. Surprisingly, we noticed that CD86-expressing cells divided more than CD80^+^ CD86^-^ or CD80^-^ CD86^-^ T cells (**Figure 2B**) indicating that CD86 was associated with a tendency to proliferate further than CD80^+^ cells. While CD80 was predominantly upregulated on cells that underwent 1–3 divisions, CD86 steadily increased as T cells progressed through rounds of divisions (**Figure 2C**). This suggests that CD86 is a marker of more expanded cells and correlated with replication index (**Figure 2C**).

To further elucidate how TCR and CD28 costimulatory signals affect CD80 and CD86 expression CTV-labelled CD4^+^CD25^-^ T cells were stimulated with cross-linked anti-CD3 or anti-CD28 antibodies alone and T cells were stained for CD80 and CD86 expression. Surprisingly, we found that CD80 and CD86 upregulation on divided cells were both driven by strong CD28 costimulation, and that TCR stimulation in the absence of CD28 had limited impact on expression of these ligands (**Figure 2D**). We have previously demonstrated that CD28 stimulation induced higher levels of CD25 and pSTAT5 phosphorylation (21). Therefore, to exclude the possibility that the observed effect might be due to limited IL-2 production following TCR stimulation, we also stimulated T cells in the presence of IL-2. Nonetheless, addition of IL-2 did not rescue CD80 and CD86 upregulation by TCR activation alone demonstrating that CD28 costimulation is required for induction of CD80 and CD86 on T cells.

Finally, we investigated whether naive and memory T cells have equal capacity to induce CD80 and CD86 upon activation. Purified CTV-labelled naive and memory T cells were stimulated with anti-CD3/anti-CD28 antibody-coated beads for five days and divided cells were analysed for CD80 and CD86 expression. We found that both CD80 and CD86 are predominantly expressed by memory T cells (**Figure 2E**).

CD28 costimulation appeared critical for CD80 and CD86 expression, resulting in T cells expressing CD80, CD86 or both (**Figures 2A–D**). Furthermore, we noted that despite gating only on activated and proliferating cells, there were 40–60% of cells not expressing these ligands, indicating that CD80 and CD86 are not equally expressed on all activated T cells. Therefore, we sought to characterize factors that differentiated towards either CD80^+^ or CD86^+^ T cells and their functional differences.

### Activated CD86^+^ T Cells are IFN-γ Expressing mTOR-Dependent Cells

**Figure 3:**
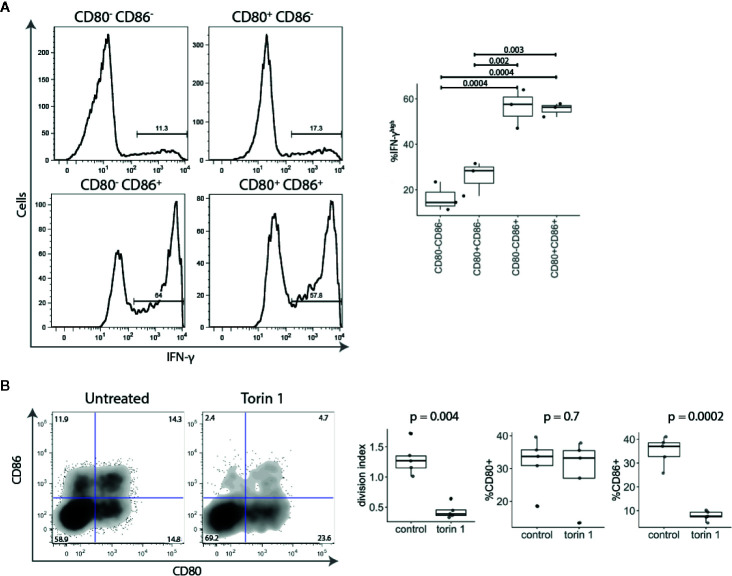
Activated CD86^+^ cells express high levels of IFN-γ and are mTOR-dependent. **(A)** Five days post stimulation, T cells were sorted into four populations based upon CD80 and CD86 expression, and restimulated with PMA and Ionomycin. All four populations of cells were gated on the same level of division. Representative histograms and collated data of percentage of IFN-γ^high^ T cells are shown. P-values were calculated using one-ANOVA. **(B)** Cell trace Violet labelled CD4^+^CD25^-^ T cells were stimulated with anti-CD3/anti-CD28 beads for five days in the presence or absence of mTOR inhibitor Torin 1 (25 nM). FACS plot of CD80 and CD86 expression in gated divided (CTV^low^) T cells. Graphs show effect of Torin 1 on cell division (represented with division index), CD80 and CD86 expression. P-values were calculated using unpaired two-tailed T-test.

Given that CD86^+^ T cells were found amongst highly proliferating activated conventional T cells (**Figures 2B, C**), we examined the expression of the effector cytokine IFN-γ. Stimulated conventional T cells were sorted into four populations based upon CD80 and CD86 expression and tested for IFN-γ production. We found that CD86 expressing T cells (either CD80^-^ CD86^+^ or CD80^+^ CD86^+^) expressed significantly more IFN-γ than CD80^+^ CD86^-^ or CD80^-^ CD86^-^ T cells (**Figure 3A**).

It has recently become clear that the level of mTOR activation is important for induction of the effector phenotype and IFN-γ production, and that in the absence of mTOR activity, T cells preferentially differentiate into Tregs (22–24). We therefore tested the effect of the mTOR inhibitor Torin 1 on CD80 and CD86 expression. As shown in **Figure 3B**, Torin 1 strikingly reduced the frequency of CD86^+^ T cells but had little effect on CD80^+^ subset. This was accompanied by reduction in proliferation, further demonstrating that CD86 is predominantly expressed on highly proliferative effector cells. Together, these data show that CD86^+^ cells are highly divided, IFN-γ producing, cells that require mTOR activity suggesting that CD86 is preferentially expressed on effector T cells. In contrast, CD80 single-positive cells produced little IFN-γ and were Torin insensitive highlighting their potential relationship to Treg.

### TGF-β and Vitamin D Induce CD80 Expression on T Cells

**Figure 4:**
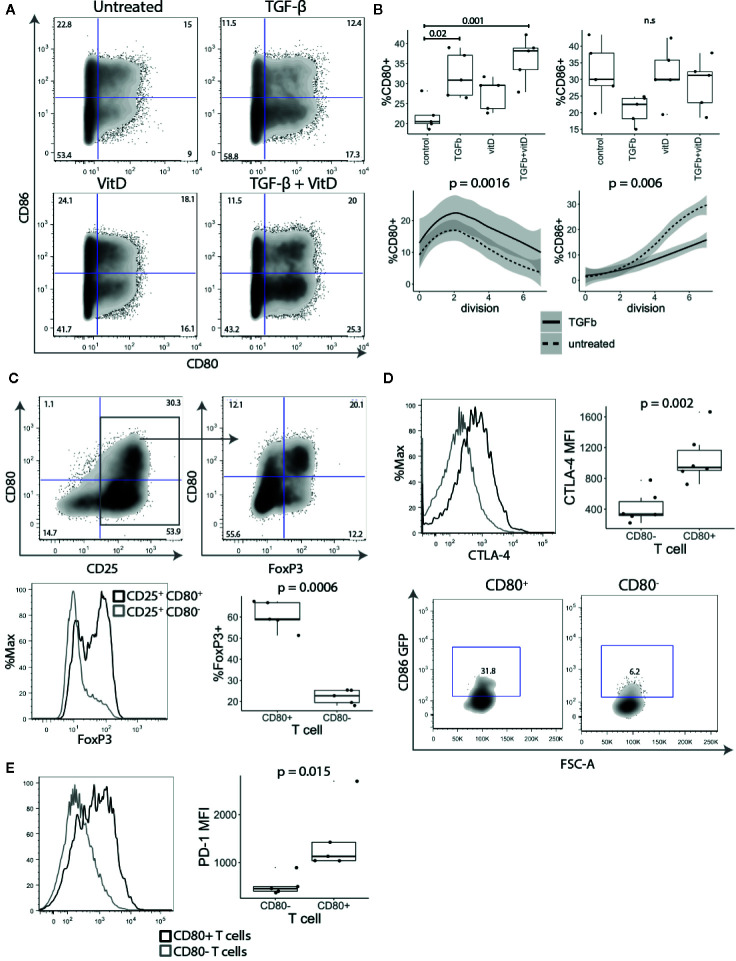
TGF-β and vitamin D upregulate CD80. **(A)** Treg depleted CD4^+^CD25^-^ T cells were stimulated with CD3/CD28 beads in presence of 1 ng/ml TGF-β, 10 nmol 1,25-Dihydroxyvitamin D3 (vitD) or TGF-β together with vitD. Plots show representative CD80 and CD86 staining. **(B)** Top panel shows the percentage of CD80 and CD86 expressing cells across different cell culture conditions. Bottom panel shows the percentage of CD80 and CD86 expressing cells across different divisions stimulated in presence or absence of 1 ng/ml TGF-β. Data points represent individual donors, P-values were calculated using one-ANOVA. n.s., not significant. **(C)** CD4^+^CD25^-^ T cells were stimulated with anti-CD3/anti-CD28 beads in the presence of 1 ng/ml TGF-β. Five days post stimulation, beads were magnetically removed and cells were rested for two days in 100 U/ml of IL-2. Representative FACS plots and graph for percentage of FoxP3 expressing cells in gated CD25^+^CD80^+^ and CD25^+^CD80^-^ T cells. P-values were calculated using paired two-tailed T-test. **(D)** Representative histogram (gated on divided T cells) and collated data of cycling CTLA-4 (37°C) median fluorescence intensity (MFI). Five days post stimulation T cells were cocultured with CHO-CD86 GFP for 6 h in 1:1 ratio in the presence of 20nM Bafilomycin A1 to measure transendocytosis. P-values were calculated using paired two-tailed T-test. **(E)** Representative histograms and collated data of percentage of PD-1 expression in CD80^+^ and CD86^+^ T cells. P-values were calculated using paired two-tailed T-test.

Since CD28 costimulation is known to play an important role in Treg biology and CD80 was observed on *ex vivo* Tregs, we investigated how stimulation with TGF-β and 1,25-dihydroxyvitamin D3 (vitD) influenced the expression of CD80. Both TGF-β and vitD have been reported to induce regulatory phenotype in conventional T cells (25–27). Treg depleted CD4^+^CD25^-^ T cells were stimulated with CD3/CD28 beads in presence of 1 ng/ml TGF-β, 10 nmol vitD or TGF-β together with vitD. This revealed that addition of TGF-β markedly increased the percentage of CD80^+^ T cells. The effect was particularly pronounced in T cells activated in the presence of the combination of TGF-β and vitD (**Figures 4A, B**). In contrast, TGF-β reduced the percentage of CD86^+^ T cells although this was not statistically significant. Further analysis showed that the observed effects of TGF-β on CD80 and CD86 expression were independent of T cell division (**Figure 4B**).

Given that TGF-β is known to be important in Treg maintenance and function (28) and it induced CD80 expression we further investigated the link between CD80^+^ cells and FoxP3 expression. As shown in **Figure 4C**, FoxP3 was strongly enriched amongst CD25^+^ CD80^+^ T cells, whilst CD25^+^ CD80^-^ T cells included a much lower frequency of FoxP3^+^ cells (**Figure 4C**), despite all cells proliferating and expressing equivalent CD25. Thus, these data suggest that CD80-expressing T cells have features consistent with thymic and induced Treg and that CD80 may be a useful surface marker for such cells.

To examine whether CD80^+^ T cells may have regulatory capacity, we assessed CTLA-4 expression, which is known to play a major role in Treg mediated suppression (29–31). CD4^+^CD25^-^ T cells were stimulated for five days with anti-CD3/anti-CD28 beads in the presence of TGF-β and stained for CTLA-4 expression. CD80^+^ T cells had both a higher percentage of cells and more importantly approximately three-fold higher level of CTLA-4 expression than activated CD80^-^ T cells (**Figure 4D**). To compare their CTLA-4-mediated regulatory capacity, CD80^+^ and CD80^-^ T cells were also co-cultured with CHO-CD86GFP to assess the capture of ligands by transendocytosis. It has previously been shown that the level of cycling CTLA-4 is directly correlated with the amount of ligand removal from APCs and therefore the degree of immune suppression (8, 32, 33). As expected CD80^+^ T cells, which had high levels of CTLA-4 acquired more CD86-GFP than equivalently activated cells lacking CD80 (**Figure 4D**). In addition to CTLA-4, we also observed that another immunoregulatory protein PD-1 was more highly expressed on CD80^+^ T cells (**Figure 4E**). Collectively these experiments suggest that CD80^+^ T cells have the properties of induced regulatory T cells with high levels of functional CTLA-4 expression along with Foxp3.

Together these results demonstrate that the CD80 single positive cells proliferate less than CD86 expressing cells, and express Foxp3, high CTLA-4 and low level of IFN-γ.

### T Cell Expressed CD80 Endogenously Interacts With CTLA-4

**Figure 5:**
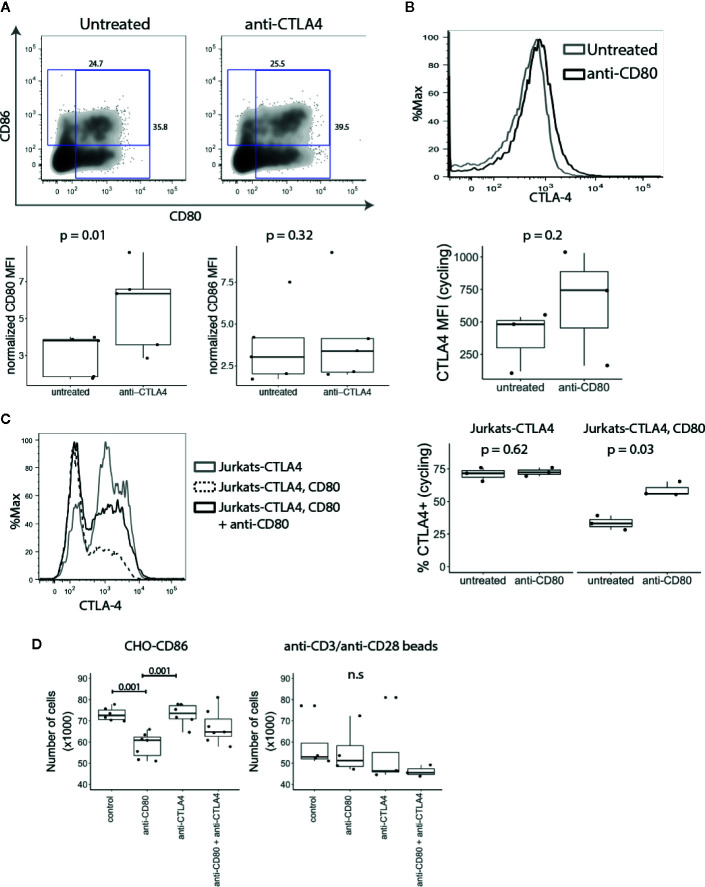
T cell expressed CD80 inhibits CTLA-4 availability and promotes Treg growth. **(A)** CD4^+^CD25^-^ T cells were stimulated with CD3/CD28 beads in presence of anti-CTLA-4 and the expression of CD80 and CD86 analysed five days post stimulation by flow cytometry. Representative FACS plots and pooled data for percentage of CD80 and CD86 expressing cells in gated divided cells are shown. P-values were calculated using paired two-tailed T-test. **(B)** CD4^+^CD25^-^ T cells were stimulated for five days with CD3/CD28 beads. On day four, anti-CD80 was added to culture overnight, and CTLA-4 expression was measured by flow cytometry the following day. Representative histogram and collated data of CTLA-4 expression in the presence or absence of anti-CD80. P-values were calculated using paired two-tailed T-test. **(C)** Jurkat-CTLA-4 cells were transduced with CD80GFP and the level of cycling CTLA-4 was examined by flow cytometry. Cells were cultured overnight in the presence or absence of anti-CD80 where indicated. Representative histogram and collated data of CTLA-4 expression. P-values were calculated using paired two-tailed T-test. **(D)** Expanded Tregs were restimulated with CHO-CD86 or anti-CD3/anti-CD28 beads in the presence or absence of anti-CD80, anti-CTLA-4 or both. Boxplots show the total number of Tregs following treatment. P-values were calculated using one-way ANOVA. n.s., not significant.

Since CTLA-4 removes CD80 and CD86 from APCs by the process of transendocytosis (10), we explored whether T cell-expressed CD80 and CD86 might interact with CTLA-4 endogenously. CD4^+^CD25^-^ T cells were stimulated with anti-CD3/anti-CD28 beads in the presence or absence of anti-CTLA-4. We predicted that if CTLA-4 binds to CD80 and CD86 endogenously, blocking CTLA-4 would result in increased levels of CD80 and CD86 on the T cell surface. As shown in **Figure 5A**, blocking CTLA-4 resulted in a significant increase in the level of CD80, while the level of CD86 was not affected suggesting that CD80 is the dominant interacting ligand.

Conversely, to test the impact of anti-CD80 on CTLA-4 we stained CTLA-4 on activated conventional T cells at 37°C to detect CTLA-4 available *via* the cell surface. Similarly, to blocking CTLA-4, when CD80 was blocked we observed a higher amount of cycling CTLA-4, albeit the effects were modest (**Figure 5B**). In addition to studying the influence of CD80 expression on CTLA-4 cycling, Jurkat-CTLA-4 cells were transduced with CD80 and we measured CTLA-4 at 37°C. Strikingly, these results demonstrated that expression of CD80 almost completely abrogated detection of cycling CTLA-4 (**Figure 5C**). More importantly, blocking CD80 for 2h, disrupted the CTLA-4 interaction between CD80 and restored CTLA-4 detection. Together these data indicated that CD80 profoundly influenced the detection of CTLA-4 by either reducing the ability of CTLA-4 to traffic to the plasma membrane or by preventing antibody binding. In either case functional CTLA-4 activity was clearly inhibited by CD80.

Since Tregs require CD28 signals for proliferation and survival (34, 35), decreased CTLA-4 expression enhances Treg proliferation (8, 30). We therefore reasoned that if intrinsic expression of CD80 on T cells impacts CTLA-4 availability this may regulate Treg numbers. We therefore expanded Tregs *in vitro* using anti-CD3/anti-CD28 beads, IL-2 and Rapamycin. After the second restimulation, anti-CD3/anti-CD28 beads were removed and T cells were stimulated with CHO-CD86 or anti-CD3/anti-CD28 beads for 24h in the presence of anti-CD80, anti-CTLA-4 or both. Strikingly, the number of Tregs stimulated with CHO-CD86 was significantly reduced by blocking CD80 (**Figure 5D**). Moreover, the effect of CD80 on Treg numbers was dependent on the CTLA-4, as blocking CTLA-4 abrogated its effect. In addition, when we blocked CTLA-4 alone we did not observe any effects. Together this suggests that CD80 functions by engaging CTLA-4 and disabling it. Accordingly, when CD80 is blocked, CTLA-4 is free to interact with CD86 on APCs, impairing their ability to deliver CD28 signals. Importantly, when Tregs were restimulated with anti-CD3/anti-CD28 beads we did not observe the effect of anti-CD80 since Treg numbers are not dependent on CD28-CD86 interactions in this setting and therefore outside the control of CTLA-4. Taken together our data show that intrinsic CD80–CTLA-4 interaction controls Treg numbers in settings where stimulation is driven by CD28-ligand interaction.

## Discussion

It is well established that CD80 and CD86 expressed on antigen presenting cells play a role in providing costimulation to T cells *via* ligation of CD28 (4, 5, 36). In addition, both ligands can also bind to the inhibitory receptor CTLA-4, which plays an important role in regulating T cell responses. Whilst these costimulatory and inhibitory functions are well recognised in the context of antigen presentation, CD80 and CD86 are also expressed on T cells in some circumstances (13–16), yet relatively little progress has been made in understanding their significance.

Given previous data showing that ligands can be physically acquired based on expression of CD28 and CTLA-4 (10, 17–19) we establish here that transfer of ligand from APCs to T cells did not account for T cell ligand expression. Our data also show that T cells express CD80 and CD86 in various combinations as a consequence of activation, with expression predominantly seen on activated, proliferating CD45RA negative cells. This occurs in settings where ligand acquisition from the APC is not possible thereby demonstrating that T cells intrinsically express CD80 and/or CD86 in response to stimulation, supported by analysis of publicly available gene expression data. Our data further indicate that CD28 engagement, even in the absence of TCR signaling, is important for CD80 and CD86 upregulation and that T cells stimulated solely by engaging TCR do not express these ligands. Accordingly, the expression of CD80 and CD86 on T cells seems likely to be influenced by the balance between TCR and CD28 signaling at the outset of a T cell response.

A further conclusion from our work is that CD80 and CD86 expression reflects differences in underlying T cell phenotype. Firstly, FoxP3, the master regulator of Treg differentiation (37, 38), is highly enriched amongst CD80^+^ T cells suggesting that these cells contain an inducible Treg subset. Moreover, conditions known to induce FoxP3, such as TGF-β and CD28 engagement (26, 39), also promote the expression of CD80. The fact that FoxP3 promoting conditions are reflected by changes in CD80 expression indicates a clear link between CD80 and FoxP3 expressing T cells. Whilst, it is not possible in the present studies to determine whether CD80 simply marks FoxP3^+^ cells or whether it plays a role in their induction, our data do support a role for CD80 in the expansion of cells with a Treg phenotype. Interestingly, it has been reported that CD80-deficient T cells are responsible for more aggressive GVHD responses in mice, consistent with a possible role in Treg function (40).

In contrast to CD80 expression on FoxP3^+^ cells, CD86 appears to mark T cells that are highly proliferative and producers of IFN-γ, indicating that CD86^+^ T cells may represent a population of activated effector T cells. Notably, single positive CD80^-^ CD86^+^ T cell subsets showed the clearest distinction with regard to IFN-γ expression, whereas CD80^+^ CD86^+^ double positive T cells appear to exhibit properties of both single positive populations, suggesting they represent an intermediate differentiation state. One striking finding relating to CD86 expression was that inhibition of mTOR had a dramatic effect on CD86^+^ cells but did not affect CD80+ cells. This is reminiscent of various studies on the impact of manipulating mTOR in mice (41) where mTOR deficiency disrupted the ability of T cells to make Th1 and other effector lineages but left Treg differentiation intact. Moreover, culture of Treg for therapeutic purposes is frequently performed in the presence of the mTOR inhibitor Rapamycin, indicating that Treg are insensitive to blockade of this pathway (42). This is in line with our observation that CD80^+^ cells also express Foxp3 and are insensitive to mTOR inhibition. In contrast, CD86 expression, which appears to be more selective for differentiation of effector T cells, is acutely sensitive to mTOR inhibition.

In general, the differential functions of CD80 and CD86 are the least well-understood aspect of the CD28/CTLA-4 system. Studies using knockout mice suggest that CD86 may be the more dominant CD28 ligand *in vivo* (43) whereas *in vitro* studies comparing the ligands align with affinity data and suggest that CD80 is a robust CD28 and CTLA-4 ligand (44, 45). These outcomes are all likely to be heavily influenced by the timing and expression level of each ligand, the types of APC present and the presence/absence of Treg. Thus, at present there is little consensus on the roles of CD80 and CD86 other than it is clear both can stimulate *via* CD28 and both interact with CTLA-4 and do so with known, variable affinities (9). The differential expression of CD80 and CD86 on regulatory and effector-like T cells now adds further insights into our understanding CD80 and CD86 function.

One intriguing observation is that Treg cells appear to specifically express the highest affinity CTLA-4 ligand, CD80, whilst at the same time relying on CTLA-4 for suppressive function (10, 29). There are several plausible explanations for this observation. CD80 might play a role in fine-tuning T cell regulation, by limiting the actions of CTLA-4, thereby allowing CD28 better access to its ligands. It is clear that ligation of CD28 is important to Treg homeostasis (34, 35). Consequently, genetic mutations that reduce CTLA-4 expression actually promote the expansion of Treg (8, 30). Since CTLA-4 on Treg restricts expansion due to competition for CD28 signals, expressing CD80 intrinsically in Treg therefore appears to blunt the competitive influence of CTLA-4 and promote Treg survival. In line with this, we demonstrated that the proliferation of Tregs stimulated with CD86 was reduced by blocking CD80, supporting the idea that blocking CD80 subsequently allows CTLA-4 to more effectively target CD86. Accordingly, the effect of anti-CD80 was dependent upon the function of CTLA-4, and simultaneously blocking CTLA-4 prevented the anti-CD80 induced reduction in Treg numbers. In line with this, we found that CD80 reduced availability of CTLA-4 by either affecting CTLA-4 trafficking to the plasma membrane, or by preventing antibody binding. The ability of CD80 to perform these functions likely relates to its unusually high avidity for CTLA-4 (9). However, there are a number of additional possibilities including the formation of non-dissociating CTLA-4–CD80 complexes due to the unique nature of CD80–CTLA-4 dimer-dimer interactions (46). In addition, altered trafficking of CTLA-4 directly due to CD80 binding also remains a possibility. From a disease perspective, it is notable that CD80 has been associated with a number of autoimmune diseases such as systemic lupus erythematosus (47) primary biliary cholangitis (48) and multiple sclerosis (49) Furthermore, unlike CD86 it also interacts with the PD-1 ligand PD-L1 indicating additional discrete regulatory functions for CD80 (50). Our data therefore suggest new contexts in which CD80 could be implicated in autoimmune diseases and cancer.

Overall, the data presented here provide evidence that CD80 and CD86 are differentially expressed on T cells with distinct functional profiles and strongly influenced by the conditions of T cell activation. Whilst the precise functional impacts of expression of these ligands still remain to be fully explored, the data presented provide further evidence that CD80 and CD86 are likely to play distinct roles in the regulation of T cell responses.

## Data Availability Statement

The original contributions presented in the study are included in the article/supplementary materials; further inquiries can be directed to the corresponding author.

## Ethics Statement

The studies involving human participants were reviewed and approved by the UCL Research Ethics Committee. The patients/participants provided their written informed consent to participate in this study.

## Author Contributions

BS and DMS conceived and designed the project. BS, LEJ, AK, DHG, TZH, NH, DJ, and BR carried out the experimental work. BS, LEJ, AK, DHG, TZH, NH, DJ, BR, GMH, and DMS interpreted the results. BS and DMS wrote the manuscript. All authors contributed to the article and approved the submitted version.

## Funding

LEJ was funded by Arthritis Research UK grant 19364, AK was funded by the Wellcome Trust (20478/Z/16), BR was funded by BBSRC grant BB/M009203/1, DJ was funded by a BBSRC iCASE studentship, CW was funded by Arthritis Research UK, grant 21147, NH was a recipient of a Wellcome Trust Clinical PhD studentship award (110297/Z/15/Z). This research was supported in part by National Institutes of Health Research (NIHR) University College London Biomedical Research Centre funding to TZH and the NIHR Rare Disease Translational Research Collaboration. DMS is a recipient of a Wellcome Trust Investigator Award (20478/Z/16).

## Conflict of Interest

The authors declare that the research was conducted in the absence of any commercial or financial relationships that could be construed as a potential conflict of interest.
